# The current state of digital manikins to support pain self-reporting: a systematic literature review

**DOI:** 10.1097/PR9.0000000000001274

**Published:** 2025-05-06

**Authors:** Syed Mustafa Ali, Danielle C. Mountain, Rebecca R. Lee, Darcy Murphy, Alessandro Chiarotto, David C. Wong, William G. Dixon, Sabine N. van der Veer

**Affiliations:** a Division of Informatics, Imaging and Data Science, Manchester Academic Health Science Centre, The University of Manchester, Manchester, United Kingdom; b National Institute for Health and Care Research (NIHR) Applied Research Collaboration—Greater Manchester (ARC-GM), Manchester, United Kingdom; c Centre for Musculoskeletal Research, Division of Musculoskeletal and Dermatological Sciences, Manchester Academic Health Science Centre, The University of Manchester, Manchester, United Kingdom; d Manchester Centre for Health Psychology, Division of Psychology and Mental Health, University of Manchester, Manchester, United Kingdom; e NIHR Manchester Biomedical Research Centre, Manchester University NHS Foundation Trust, Manchester, United Kingdom; f Department of General Practice, Erasmus MC, University Medical Center, Rotterdam, the Netherlands; g The Leeds Institute of Health Sciences, The University of Leeds, Leeds, United Kingdom; h Northern Care Alliance NHS Foundation Trust, Salford, United Kingdom

**Keywords:** Chronic Pain, Pain measurement, Digital pain manikins, Patient-generated health data, Systematic review

## Abstract

This systematic literature review aimed to explore the current state of digital manikins to support pain self-reporting and to explore the extent to which manikins had progressed along the translational pathway. We systematically searched six electronic databases using a combination of key words and MeSH terms for “pain” and “manikin” to identify original studies in English that used a digital pain manikin for collecting information from adults with any condition. We extracted and descriptively synthesised data on the characteristics of studies and digital pain manikins and mapped them to a stage of the translational pathway (ie, design, testing, metric validation, and diffusion). We screened 6,189 articles, of which we included 104. Articles reported on 31 unique digital pain manikins. Most studies were conducted in Europe (n = 60; 58%), recruited people with pain/painful conditions (n = 69; 66%) from clinical settings (n = 67; 64%). Of the 31 manikins, the majority were two-dimensional (n = 21; 68%) with a front and back body view (n = 18; 58%) and allowed users to draw their pain on any area of the manikin (n = 23; 74%). Most manikins were still in the development and testing stages (n = 23; 74%). Only eight (26%) had progressed fully through the translational pathway, taking between 7 and 20 years to go from early development to diffusion. We found a substantial number of mostly two-dimensional digital pain manikins reported on in the last decade. However, most were still at early stages of the translational pathway, with only few having progressed through to diffusion into research and health care settings.

## Abstract 2

Supplemental Digital Content is Available in the Text.

The systematic review of scientific literature identified 31 digital pain manikins, out of which only a few progressed to be used as a pain self-reporting tool.

## 1. Introduction

Chronic pain is a global public health problem, the prevalence of which is increasing every year.^47^ Chronic pain significantly affects physical, psychological, and social aspects of life for people living with this condition.^12,15,46^ Chronic pain is now considered a long-term condition in its own right as well as a condition that can be secondary to underlying disease processes (eg, rheumatoid arthritis).^35,39^

Pain is a subjective experience, explaining why self-reporting by people living with chronic pain is important for assessing pain and evaluating the effectiveness of treatments.^14,20,40^ The Initiative on Methods, Measurement, and Pain Assessment in Clinical Trials recommended (temporal changes in) pain intensity, quality, location, and bodily distribution as core domains for the assessment of pain treatment effectiveness.^17^ Pain aspects, such as pain intensity, interference (eg, quality of life and activities of daily living) are recommended core domains for research and clinical practices.^11^ However, currently available questionnaire-based pain assessment tools often do not cover these domains sufficiently^21,26^ and have adoption barriers (eg, lack of validation, time needed for completion, language barriers).^13,14,25^

Pain manikins, also called pain body maps or pain drawings, may address some of the limitations of self-reporting questionnaires.^12,27,28,31,32,47^ They are particularly suitable for measuring pain location and location-specific pain aspects. The first pen-and-paper version appeared in the 1940s,^38^ and the first digital version was developed in the 1990s.^36^ Since then, many digital pain manikins have been developed, but the diffusion of digital pain manikins into health care and research practices has been slow. Although there have been significant methodological developments of digital pain manikins,^43^ a 2022 review showed that they only started to be used more widely as research data collection tools in published studies from 2017 onwards^4^; 25 years or more after the first digital manikin was developed.^36^ Furthermore, reviews of digital pain manikins found that most manikins did not come with metrics to summarise manikin reports.^3,4^ This in turn may result in manikins not meeting the standards for widespread use and, therefore, not progressing along the translational pathway. However, it remains largely unclear what explains this limited adoption of digital manikins.

Therefore, this systematic literature review aimed to explore the current state of the development and adoption of digital pain manikins. Specific objectives were to (1) identify and characterise published studies reporting on the development, evaluation, or use of a digital pain manikin; (2) characterise these digital pain manikins; and (3) explore the extent to which the manikins progressed along the translational pathway.

## 2. Methods

We used the Preferred Reporting Items for Systematic Reviews and Meta-Analyses guidelines^37^ for the reporting of our results. The systematic review protocol was registered on PROSPERO (an international database of prospectively registered systematic reviews in health and social care) before commencing the review.^2^ The protocol is linked to two other publications: one aimed at the data science community with a focus on manikin data analysis approaches^34^ and one conference paper reporting on studies excluded for the current review because they used a manikin for data collection but without there being reports of the manikin's development or validation.^4^

### 2.1. Search strategy

We systematically searched Medline, CINAHL and Embase via Ovid, and Scopus on November 3rd, 2020, and we updated our search results on August 23rd, 2023. In addition, we searched the IEEE Xplore digital library and ACM Digital Library for literature in the fields of engineering and technology. We used a combination of key words and MeSH terms related to “pain” and “manikin” (see full search syntax in appendix A, http://links.lww.com/PR9/A306). We did not limit our search to publication date. Finally, we manually searched reference lists of included articles and of those on a relevant topic that had been excluded based on study or publication type (eg, reviews) to further identify eligible articles.

### 2.2. Study selection

#### 2.2.1. Inclusion and exclusion criteria

Studies were deemed relevant if they were published in English and met the following criteria:

1. *Study population*: studies with adult participants of 16 years or older with personal experience of pain, as well as adult healthy volunteers. The latter enabled us to identify digital manikins in early stages of development;
2. *Intended manikin users*: adults with personal pain experience, thereby excluding manikins aimed at supporting pain recording by health care professionals or researchers;
3. *Digital pain manikin*: any human-shaped figure that facilitated interactive self-reporting of pain in any part or location of the body^44^ on a digital device, eg, a smartphone, or a desktop or tablet computer. This included two- and three-dimensional body shapes, as well as manikins of a specific body part (eg, head manikin for migraine-related pain). We also included studies that aimed to develop a digital manikin and used a paper-based manikin to gather end user feedback to inform the design of the initial prototype. For studies where we were unable to confirm whether the manikin was (planned to be) digital, we contacted their corresponding authors (with a maximum of two reminders) and excluded studies if we did not receive a reply. We also excluded studies that scanned paper-based manikins into digital format, as well as those that used static, illustrative manikins for educational or communication purposes. For preparing a list of unique manikins, we identified unique manikins across studies based on the manikin's name but, if no name was mentioned, we derived this from contextual information (eg, authors list, manikin's description);
4. *Outcome of interest related to manikins*: any aspect of chronic, acute, simulated, or induced pain collected using the digital manikin, eg, pain location, location-specific pain intensity, location-specific pain quality. We excluded studies that used a manikin to assess other location-specific disease aspects (eg, tender and swollen joints in people with rheumatoid arthritis).
5. *Study type*: studies that reported on any of the stages in manikins' translational pathway (see Table 1). This included studies that aimed to develop, test, or validate a digital pain manikin and manikin-derived metrics, including proof-of-concept and feasibility studies. As we aimed to understand manikins' progression through the translational pathway, we excluded studies if they reported using a digital manikin for collecting pain self-reports (ie, diffusion stage) but without describing or referencing any work related to previous stages.
6. *Publication type*: peer-reviewed journal and full conference papers, excluding grey literature, preprints, protocols, reviews, commentaries, editorials, and conference abstracts.

**Table 1:** Box 1: Stages of the translational pathway (Sendak et al.,^42^) and their description.

Stage	Description	Examples
Stage 1: Design	Manikin design, early development, and testing in controlled settings	Studies gathering requirements and feedback (eg, on usability, acceptability, perceived usefulness) from people living with pain and other stakeholders (eg, clinicians) for designing and developing digital manikin for pain self-reporting; this included studies testing manikins in laboratory/controlled settings (eg, in a usability laboratory or operating theatre supervised by a researcher)
Stage 2: Testing	Manikin testing in real-world settings	Studies assessing feasibility and acceptability of using a digital manikin unsupervised (eg, at home)
Stage 3: Metric validation	Development and validation of manikin-derived metrics	Studies gathering requirements from people living with pain and others involved in pain monitoring/management regarding useful and meaningful manikin-derived metrics; assessing the measurement properties of these metrics (eg, validity, reliability, responsiveness); and developing advanced analytical approaches of summarising manikins visually or numerically
Stage 4: Diffusion	Diffusion of manikin for pain self-reporting in health care or research settings	Studies using a digital pain manikin in a health care or research settings, either alone or in combination with other patient-reported outcome measures. We also assigned manikins to this stage, if they appeared in our web and app store search, regardless of whether they had any published studies reporting on their diffusion

### 2.3. Screening process

After removing duplicates from the database searches, two reviewers independently screened each title and abstract. One reviewer (S.M.A.) screened all titles/abstracts, with four reviewers each screening a proportion (R.R.L., D.C.M., D.M., S.N.v.d.V.). For potentially relevant studies, the same reviewer pair retrieved and assessed the full text. Reviewer pairs met regularly to discuss the screening results and reach consensus on disagreements. Disagreements were discussed with a third reviewer, if needed. Reasons for exclusion were recorded for the full-text screening stage only.

### 2.4. Data extraction and synthesis

We used published reviews^3,43^ to develop the data extraction templates, which we pilot-tested for clarity and completeness. In line with the review's aim to describe the current state of play of digital pain manikins rather than their impact on outcomes, we did not extract information to assess the risk of bias or quality of studies.

We extracted information on:

1. Study characteristics (objective 1): year of publication, country, study settings, study population type, and sample size;
2. Digital pain manikin characteristics (objective 2): name of manikin or programme; data collection device, number of dimensions, number of views of manikin, manikin personalisation features; method for pain location recording; and manikin-recorded pain aspects;
3. Translational stages (objective 3): for each study, we determined to which of the four stage(s) of the translational pathway it pertained: design (stage 1); testing (stage 2); metric validation (stage 3); or diffusion (stage 4). A single study could pertain to more than one translational stage, depending on its objectives. Table 1 describes and illustrates the stages, which we adapted from a translational pathway developed for machine learning products.^42^ For all manikins with a name, we checked whether they were included in a previous manikin app review^3^ and conducted a web and app store search to see if they were publicly available. For manikins that mapped to more than one translational stage, we reported on the time between earliest and latest stage; for stages with one than one study, we used the year of publication of the earliest study.

Four reviewers (S.M.A., D.C.M., D.M., S.N.v.d.V.) extracted data independently and in duplicate (ie, in pairs) and solved discrepancies through discussion and then synthesised extracted data narratively per objective.

## 3. Results

**Figure 1.**
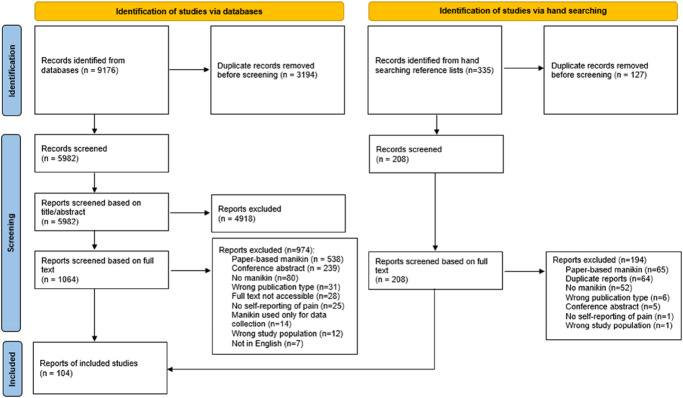
PRISMA flow diagram of article selection.

Figure 1 shows our searches yielded a total of 9511 records, out of which 104 studies were included. The main reason for exclusion was that the manikin was not (confirmed to be) digital.

### 3.1. Characteristics of included studies

Out of 104 included studies, the majority were published after 2018 (n = 67; 64%), conducted in Europe or North America (n = 100; 96%), and recruited no more than 150 people (n = 74; 71%) with pain/painful conditions (n = 69; 66%) in clinical settings (n = 67; 64%) (see Table 2 - see Appendix B, http://links.lww.com/PR9/A307, for study-level information).

**Table 2:** Characteristics of included studies (total n = 104).

Characteristic	No. (%)*
Publication period	
1992–2002	3 (3)
2003–2012	15 (14)
2013–2017	19 (18)
2018–2023	67 (64)
Geographical location	
Europe	60 (58)
North America	40 (38)
Asia	2 (2)
Australia	1 (1)
Multiple regions	1 (1)
Study settings	
Clinical settings	67 (64)
Nonclinical settings†	27 (26)
Mixed settings	10 (9)
Study population type	
People with pain/painful condition	69 (66)
General population or mixed study population	25 (24)
People without pain/painful conditions‡	10 (9)
Study population size	
≤50	51 (49)
51–150	23 (22)
151–300	13 (13)
>300	16 (15)
Not reported	1 (1)

* Percentages may not add up to 100% due to rounding.

† Examples of nonclinical settings are universities, research labs, football clubs, and population-level surveys.

‡ Healthy volunteers, people with amputation.

### 3.2. Characteristics of digital pain manikins

**Figure 2.**
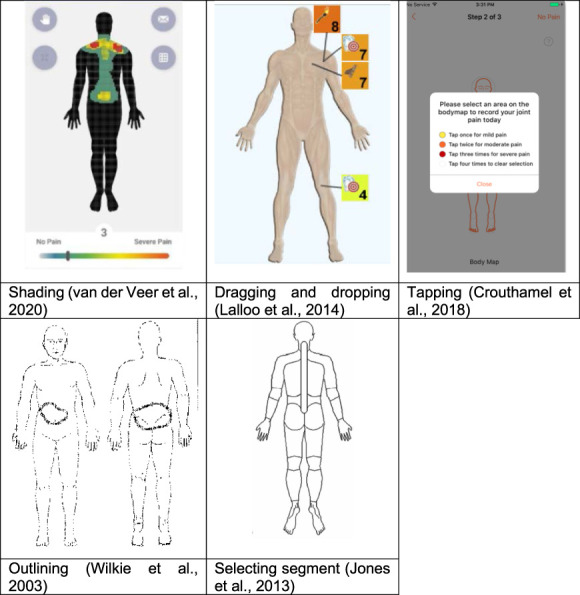
Selected manikins showing different methods of recording pain location.

The 104 included studies reported on 31 unique digital manikins (see Appendix B, http://links.lww.com/PR9/A307, for further manikin-level information). For 22 manikins, the manikin's app or system name (eg, Navigate Pain, PainDroid) or the software used to develop it (eg, SketchBook Pro, REDCap) was reported. Navigate Pain, SketchBook Pro, and Collaborative Health Outcomes Information Registry (CHOIR) were reported in 23, 14 and nine studies, respectively. Table 3 shows that most manikins appeared after 2015 (n = 21; 68%) were two-dimensional (n = 21; 68%), had a front and back body view (n = 18; 58%), and allowed users to indicate the location of their pain on any area of the manikin (n = 23; 74%). Related to the latter, Figure 2 further illustrates the different methods for pain location recording.

**Table 3:** Characteristics of digital pain manikins (n = 31) reported in included studies (n = 104).

Characteristic	No. (%)
Number of dimensions	
2D	21 (68)
3D*	10 (32)
Number of views	
Single	2 (6.5)
Two	18 (58)
Three	2 (6.5)
Four or more	9 (29)
Personalisation of body representation†	
Not reported	19 (61)
Yes	12 (39)
Method for pain recording	
Selecting any area‡	23 (74)
Selecting pre-specified areas	7 (23)
Not reported	1 (3)
Location-specific pain aspects	
Yes (eg, pain quality, intensity, depth)	16 (52)
No	15 (48)
Data collection Device	
Mobile device (eg, smartphone tablet)	11 (36)
Multiple	9 (29)
Desktop or laptop computer	6 (19)
Not reported	5 (16)
Year of first publication	
1992–2002	1 (3)
2003–2012	5 (16)
2013–2017	9 (29)
2018–2023	16 (52)

* One digital pain manikin app allowed user to upload image of their own body to report their pain.

† For example, personalisation for gender, body shape, and skin colour.

‡ Users could select any area on a manikin by either shading with finger or stylus, tapping, outlining, or drag and drop symbol or icon.

Half of the manikins (n = 16; 52%) facilitated recording of location-specific pain aspects (eg, pain quality, intensity). For example, in the Symptom Mapper, participants were asked to select the quality of their pain (by picking a pain quality descriptor from a list), its intensity (using a visual analogue scale), and its depth (eg, skin, muscle, or bone) before drawing their pain on the manikin. Participants could repeat the same procedure to report their pain for other locations.^33^ Finally, 12 manikins (39%) provided an option for personalising the manikin's body representation based on gender, while the rest of the manikins were gender neutral.

### 3.3. Stages of the translational pathway

**Figure 3.**
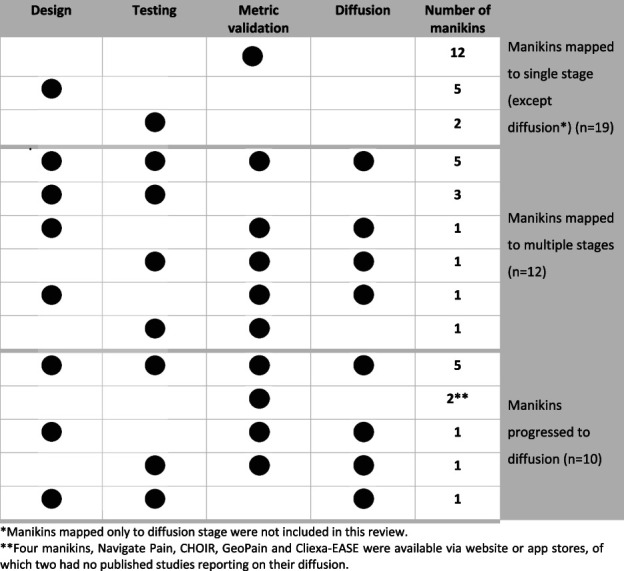
Digital pain manikins mapped to stages in translational pathway.

Figure 3 shows how many individual manikins pertained to each translational stage. Nineteen manikins pertained to a single stage (five, two, 12, and no manikins to the design, testing, metric validation, and diffusion stage, respectively). The remaining 12 manikins had reports on multiple stages. Of the four manikins accessible publicly, Cliexa-EASE was available via app store but only had report on metric validation.^24^ GeoPain, Navigate pain, and CHOIR^41^ were only available via a website where health care and research organisations could buy a subscription for a certain number of patients or participants. Only Navigate pain and CHOIR had reports on all four translational stages.

Only 10 of 31 manikins progressed to the diffusion stage, including Navigate pain, CHOIR, the SketchBook Pro manikin, Symptom Mapper, Dynamic pain drawings, PAINReportIt, the PRISMap manikin, GeoPain, Cliexa-EASE, and the manikin reported by North et al. They were used to answer questions related to chronic pain epidemiology and evaluating treatment response. Of these 10 manikins, five reported on all four translational stages, two reported only on metric validation stage, while Symptom Mapper (no report on testing), PRISMap manikin (no report on design), and the manikin reported by North et al., (no report on metric validation) all lacked reports on one of the stages. Most manikins reaching the diffusion stage (n = 10) were two-dimensional (n = 6; 60%), had two body views (n = 6; 60%), enabled gender-personalisation of manikins (n = 7; 70%), and allowed reporting of location-specific pain intensity (n = 6; 60%).

**Figure 4.**
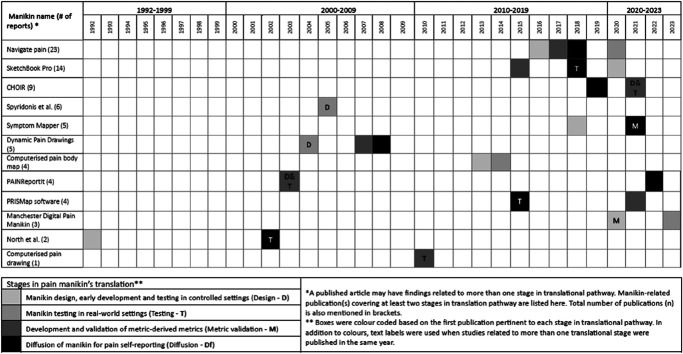
Timeline of pain manikins' progression across the translational pathway; only manikins reporting on at least two translational stages are included here.

Figure 4 summarises the timeline for how manikins with reports on at least two translational stages progressed through the translational pathway. Among the eight manikins progressing to diffusion (two manikins reported only on metric validation), it took an average of 7 years to reach this stage after the first published report, ranging from 3 years (Navigate Pain and CHOIR) to 20 (PAINReportIt). However, progression was not linear for many manikins. For example, a report on the diffusion of CHOIR^10^ was published before results related to its metric validation became available.^8^

## 4. Discussion

This review identified 104 studies reporting on 31 unique digital pain manikins. Most included studies were published after 2018 and conducted in Europe and North America with participants recruited from clinical settings. Most manikins first appeared after 2015, were gender neutral, had two dimensions with front and back views, and enabled reporting of location-specific pain aspects (eg, intensity, quality) by selecting any area directly on the manikin. Manikin personalisation was offered in several manikins but was limited to gender only. Eight manikins progressed along the translational pathway to reach the diffusion stage, taking between 3 and 20 years; two mapped to the diffusion stage were publicly available but without reports of their diffusion included in our review. One manikin was available via app stores, and three manikins were more widely available but only via subscriptions for health care and research organisations.

### 4.1. Relation to other studies

Similar to a systematic review of manikin-based apps,^3^ we found in our review that only some digital pain manikins were three-dimensional or had four views (ie, front, back, left and right), despite the first three-dimensional manikin being developed in 2008.^23^ Having only front and back views means that many current manikins may not align with users' preferences^5,6^ and suboptimally facilitate pain-related communication between patients and providers.^18^ Furthermore, women prefer a gender-specific manikin,^18,22^ but in keeping with the above-mentioned app review,^3^ we found that manikin personalisation based on gender was not implemented widely. Manikin personalisation based on body shapes and skin tones is also recommended for cross-cultural acceptability of digital pain manikins,^5^ but none of the manikins had implemented these personalisation features. This misalignment between user preferences and current manikin characteristics may affect user engagement negatively, thereby partly explaining the slow adoption and diffusion of digital pain manikins.

We excluded 14 studies that used a digital pain manikin for data collection but without describing or referencing prior testing and validation work; these were reported on separately.^4^ This indicated that most manikins reported in the scientific literature had been tested and/or validated. This is in contrast with manikin-based apps available in app stores, which often lack evaluation and involvement of end-users.^3,30^ A previous app review^3^ identified two manikins (ie, GeoPain and Cliexa-EASE), which also appeared in the current literature review. However, these only had reports on the metric validation stage,^24,29^ suggesting that these manikins were publicly available without reports on the design, testing, or diffusion stages. Moreover, GeoPain, which was previously available on app stores, is now only available via subscription. At the same time, the two digital pain manikins in our review that were rigorously designed, tested, and validated before becoming publicly available (such as Navigate Pain) have not yet made their way to app stores.

## 4.2. Limitations

This review has several limitations. First, to balance the specificity against the sensitivity of our electronic search strategy, we did not include more general terms for pain manikins, such as “pain assessment tool.” Also, we excluded studies where we could not confirm with sufficient certainty if the manikin was digital. These limitations mean that we may have missed studies and manikins, and that the numbers reported in this review may be underestimated. Second, we identified unique manikins across studies based on the manikin's name or from the contextual information (such as the author list in absence of a clear manikin name). This might have resulted in some manikins incorrectly being grouped together as a single manikin in our synthesis.

## 4.3. Implications for future development and diffusion of digital pain manikins

For manikins to be more relevant self-reporting tools for people living with pain, future developments should consider embedding three-dimensional layouts of manikins and features enabling personalisation of the body image based on users' characteristics (such as gender, body shape, skin tone). Such developments may improve capturing of pain information when codesigned with people who may face inequities in accessing and benefitting from pain services.^7^ Improving the equity of digital pain manikins will enable more diverse user engagement and enhance clinical utility of pain manikins,^1,11,18,45^ which will ultimately contribute to their wider adoption^47^ and the digital transformation of pain management services.

Developers and researchers should consider investigating and reporting on the design, testing, metric validation, and diffusion as important steps along the translational pathway. Moreover, leveraging machine learning approaches may enhance manikins' capacity to support early diagnosis and self-management, for example by automating interpretation of pain drawings to identify pain patterns or analyse the causes and effects of pain.^9,19^ This is likely to contribute to increasing the chances of a manikin's diffusion into clinical, research, and self-management practices and enhancing the usefulness and robustness of digital pain manikins as pain self-reporting tools.

## 5. Conclusions

Many new digital pain manikins have been reported on in the last decade but the majority remain two-dimensional, do not allow for personalisation, and have not made it past the validation stage on the translational pathway. Those that had been robustly designed, tested, validated, and diffused were not available on app stores, whereas the few that were available on app stores lacked reports on several translational stages. Future manikin developments should consider focusing on embedding three-dimensional body layouts and personalisation features, while reporting their findings when designing, testing, validating, and diffusing their manikin. Together, this will expedite the adoption of digital manikins as pain self-reporting tools.

## Disclosures

The authors have no conflict of interest to declare.

## Appendix A. Supplemental digital content

Supplemental digital content associated with this article can be found online at http://links.lww.com/PR9/A306, and http://links.lww.com/PR9/A307.
